# *EVA1C* Is a Potential Prognostic Biomarker and Correlated With Immune Infiltration Levels in WHO Grade II/III Glioma

**DOI:** 10.3389/fimmu.2021.683572

**Published:** 2021-06-29

**Authors:** Zhicheng Hu, Shanqiang Qu

**Affiliations:** ^1^ Department of Burn Surgery, The First Affiliated Hospital, Sun Yat-sen University, Guangzhou, China; ^2^ Department of Neurosurgery, Nanfang Hospital, Southern Medical University, Guangzhou, China

**Keywords:** *EVA1C*, immune infiltration, biomarker, glioma, microenvironment

## Abstract

**Background:**

Immunotherapy is an effective therapeutic approach for multiple human cancer types. However, the correlations between *EVA1C* and patients’ prognosis as well as immune infiltration remain obscure. Herein, we employed transcriptomic and clinical data extracted from two independent databases to systematically investigate the role of *EVA1C* in the oncological context.

**Methods:**

The differential expression of *EVA1C* was analyzed *via* TCGA and Oncomine databases. We evaluated the influence of *EVA1C* on clinical prognosis using Kaplan-Meier plotter. We then used the expression profiler to calculate stromal score, immune score, and ESTIMATE score based on the ESTIMATE algorithm. The abundance of infiltrating immune cells was calculated *via* TIMER. The correlations between *EVA1C* expression and immune infiltration levels were analyzed in two independent cohorts.

**Results:**

In patients with World Health Organization (WHO) grade II/III glioma, high *EVA1C* expression was associated with malignant clinicopathological features and poor overall survival in both cohorts. *EVA1C* expression was positively associated with immune infiltration levels of B cell, CD4+ T cell, neutrophil, macrophage, and dendritic cells (DCs). Besides, *EVA1C* expression strongly correlated with diverse immune marker sets. And the predictive power of *EVA1C* was better than that of other indicators in predicting high immune infiltration levels in glioma.

**Conclusions:**

For the first time, we identified the overexpression of *EVA1C* in glioma, which was tightly correlated with the high infiltration levels of multiple immune cells as well as poor prognosis. Meanwhile, *EVA1C* might be a potential biomarker for predicting high immune infiltration in WHO grade II/III gliomas.

## Introduction

Human brain is derived from the neural ectoderm and accounts for about half of all intracranial tumor incidences (1). In China, there are 106,207 new cases and 59,120 glioma-related deaths each year (2). Diffuse WHOII/III glioma is a lethal threat to young adults, which tends to have a wide range of genetic and transcriptional heterogeneity (3). Compared with WHOIV gliomas, the course of WHOII/III gliomas is very slow. Recent studies have mainly classified gliomas based on two genetic markers, such as isocitrate dehydrogenase (IDH) mutation and codeletion of chromosome arms 1p and 19q (codeletion) (4). In the vast majority of WHOII/III gliomas, IDH mutation is present in 84% of cases, and 1p/19q codeletion is present in 35% of cases. IDH mutation tends to occur in the early stage of gliomas (3). Although adjuvant therapeutics have improved the prognosis of glioma to some extent, the overall survival (OS) of glioma patients remains poor (5, 6). Thus, novel strategies are in urgent need for the hope to improve the unpleasing outcomes.

Immunotherapy has emerged as one of the most important therapeutic means for tumor in the past decades (7, 8). Especially, approaches targeting recognized immune checkpoints, such as anti-PD-1 and anti-CTLA4, have been approved for clinical utilization and achieved encouraging outcomes (9, 10). However, there are still some limitations in existing T cell-based immunotherapies, which are attributed to the extremely complex immunosuppressive processes of tumor microenvironment (TME) and its regulatory networks (11). Importantly, many patients didn’t respond well or acquired rapid resistance to the immune checkpoint blockers in clinical practice. Therefore, it is necessary to further explore the immunosuppressive essence and the underlying mechanism within TME (12). Existing studies have confirmed that local TME is composed of various cell types, including tumor cells, infiltrating cells and stromal cells, as well as soluble factors that support tumor growth and progression (13). TME usually confers a high degree of immunosuppression, preventing the clearance of malignant cells by immune components, which negatively impacts cancer immunotherapy (14). Therefore, seeking novel immune checkpoints and overcoming immunosuppressive processes are very critical for the improvement of effective immunotherapies against tumors.

*EVA1C* (aliases *C21orf63*), first identified in 2001, is a membrane protein encoding-gene (15). *EVA1C* protein has been found in a variety of human tissues. Kanae Mitsunaga (16) identified *EVA1C* protein possessing two repeats of putative ‘galactose-binding lectin domains’ that bind heparin. Although the role of EVA1C has not been reported in tumor, Manas Kotepui et al. reported that *ADGRL3* (*LPHN3*), an important paralog of *EVA1C* gene, was upregulated in breast cancer and was correlated with axillary lymph node metastasis (17). In addition, Inna M. Yasinska et al. found that the PKCa pathway could be activated by *FLRT3 via LPHN1*, *LPHN2* and *LPHN3* (18). Collectively, their findings indicated *FLRT3-LPHN-Tim-3-galectin-9* pathway plays a key role in escaping systemic immunosurveillance across various cancer types. However, the specific expression and function of *EVA1C*, especially in the context of immuno-oncologic interactions, remain poorly understood.

Herein, our study was aimed to identify the *EVA1C* expression and its correlation with clinicopathological factors, and survival prognosis of patients with WHO grade II/III glioma. Meanwhile, we focused on the correlation between *EVA1C* expression and abundance of immune infiltrates by immune profiles, and further investigate whether *EVA1C* could act as a new immune marker for assessing immune microenvironment of glioma patients.

## Materials and Methods

### Data Extraction

The mRNA sequencing data and clinicopathological data of all cases in this study were extracted from the Chinese Glioma Genome Atlas (CGGA, http://www.cgga.org.cn/) and The Cancer Genome Atlas (TCGA, https://tcga-data.nci.nih.gov/tcga/) databases. Patients with incomplete follow-up data were excluded. Finally, a total of 182 patients with WHO grade II/III glioma were included from the CGGA (Dataset ID: mRNAseq_325) database as the CGGA cohort, and 457 patients with WHO grade II/III glioma from TCGA database were defined as validation cohort. The detailed demographics of enrolled glioma patients in both cohorts were included in **Supplementary Tables 1** and **2**, respectively. The Estimation of Stromal and Immune cells in gliomas using Expression data (ESTIMATE) and Tumor Immune Estimation Resource (TIMER) algorithms were used to explore the immune infiltration landscapes. Since all the data are from public databases, the Ethics Committee of Nanfang Hospital granted ethical approval for the study, but waived the requirement for informed consents.

### Differential Expression of *EVA1C* in Tumors

The GEPIA (http://gepia.cancer-pku.cn/) is an online tool for dynamic analysis of gene expression profile data. GEPIA analyzed the RNA sequencing data of 9736 tumors and 8587 normal samples from TCGA and GTEx projects. The expression data of TCGA and GTEx were recalculated under the same pipeline, which can be used for very comprehensive expression analysis directly. Therefore, we employed the GEPIA webtool to examine the *EVA1C* expression profile and its correlation with patients’ prognosis. We further verified the differential expression of *EVA1C* at the mRNA level in glioma *via* the Oncomine database (www.oncomine.com). Normalized mRNA expression data for CCLE human cancer cell lines were extracted from the CCLE portal (https://portals.broadinstitute.org/ccle). The expression of *EVA1C* protein was obtained online from the Human Protein Atlas (HPA, www.proteinatlas.org).

### GO and KEGG Pathway Enrichment Analyses

To understand the potential biological functions of *EVA1C*, including molecular function, biological processes, and cellular components, we employed the DAVID database (Version 6.8, http://david.abcc.ncifcrf.gov/) to perform the GO and KEGG enrichment analyses. At first, we used the co-expression scores to obtain the top 1000 genes (19) co-expressed with *EVA1C*, which were used for subsequent functional and pathway enrichment analyses including Kyoto Encyclopedia of Genes and Genomes (KEGG) pathway and Gene Ontology (GO) analyses (*P* < 0.05 and false discovery rate (FDR) < 0.05). And the potential protein-protein interaction (PPI) networks were developed using the STRING database (Version 11; https://string-db.org/), which is an online search database for protein interaction relationship. Additionally, GO analyses were further analyzed with Coexpdia (http://www.coexpedia.org/) that was based on GEO datasets.

### Statistical Analysis

Statistical analysis was performed by SPSS (version 23.0, Corp., Armonk, NY, USA) and R programming language (Version 3.6.1). The ESTIMATE immune score and stromal score were computed by the ESTIMATE algorithm. Different immune cells infiltration levels were calculated by TIMER algorithm. The chi-square tests were performed to calculate the difference of categorical data. Spearman’s correlation analyses were used to gauge the degree of correlation between certain variables. And the survival plots were generated by the Kaplan-Meier method. Time-dependent receiver operating characteristic (ROC) curves were constructed using the R programming language. All tests were two-sided, and *P* value <0.05 was the significance threshold in this study.

## Results

### The mRNA and Protein Levels of *EVA1C* Were Upregulated in Glioma

**Figure 1:**
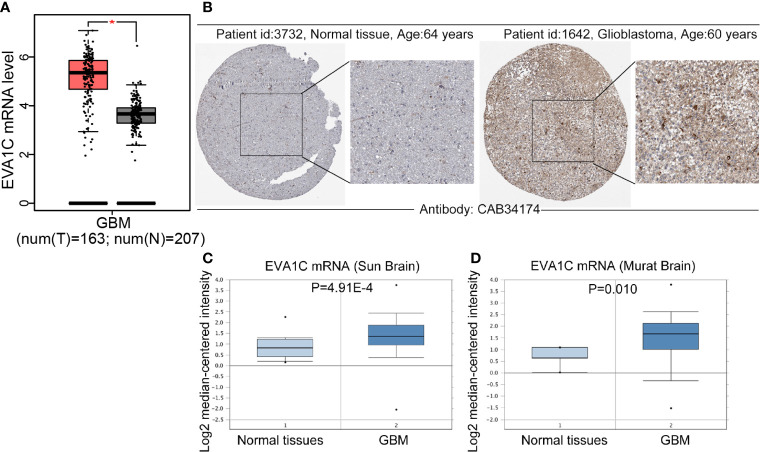
Compared with normal brain tissues, *EVA1C* expression was upregulated in glioma. **(A, B)** Compared with those in the normal brain tissues, the *EVA1C* mRNA and protein levels in GBM were upregulated. **(C, D)** The mRNA levels of *EVA1C* were upregulated in GBM (Oncomine database). T, Tumor tissues; N, Normal tissues.

Firstly, we compared the differences in *EVA1C* expression between glioma and normal brain tissues by GEPIA website, and found that the mRNA levels of *EVA1C* were upregulated in glioblastoma (GBM) (**Figure 1A**). Meanwhile, immunohistochemistry results showed that *EVA1C* protein was strongly over-expressed in GBM compared with normal brain tissues (**Figure 1B**). The upregulation of *EVA1C* mRNA in GBM was also verified in two independent cohorts (‘Sun Brain’ and ‘Murat Brain’) from the Oncomine database (**Figures 1C, D** **)**. The *EVA1C* expression in different tumors cell lines was obtained from CCLE database, it was also confirmed that *EVA1C* was highly expression in glioma cell lines (**Supplementary Figure 1A**). Additionally, we also found that, compared with normal tissues, *EVA1C* mRNA levels were higher in other cancer types including kidney renal clear cell carcinoma (KIRC), acute myeloid leukemia (LAML), pancreatic adenocarcinoma (PAAD), and thymoma (THYM) (**Supplementary Figure 1B**), and *EVA1C* protein levels were upregulated in renal cancer and pancreatic cancer (**Supplementary Figure 1C**).

### Elevated *EVA1C* Expression Correlated With Malignant Clinicopathological Features and Poor Prognosis in Patients With WHO II/III Glioma

**Figure 2:**
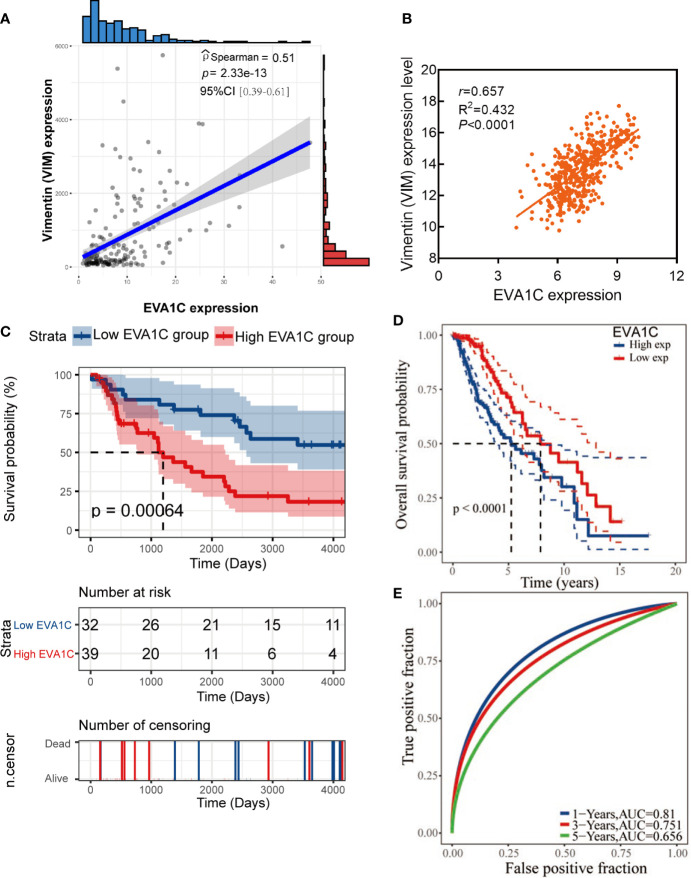
High *EVA1C* expression was associated with poor prognosis in WHO grade II/III glioma. **(A, B)** *EVA1C* expression was strongly correlated with vimentin expression in the CGGA and TCGA cohorts. **(C, D)** Patients with high *EVA1C* expression had a poor prognosis in the CGGA and TCGA cohorts. **(E)** The predictive power of *EVA1C* for predicting the 1-, 3-, and 5-year survival rate was relatively strong (TCGA cohort).

To identify the role of *EVA1C* in glioma, we statistically analyzed the correlation between *EVA1C* expression and clinicopathological features as well as prognosis in the CGGA cohort of 182 patients with WHO grade II/III glioma. The 182 patients were divided into low and high *EVA1C* expression groups based on the median value of *EVA1C* mRNA level. Results of chi-square tests revealed that high *EVA1C* expression was significantly correlated with several malignant features including WHO grade, histopathological type, IDH mutation status, and 1p/19q non-codeletion (**Table 1**, **Supplementary Figure 2**). However, the correlations were not significant between *EVA1C* expression and age, gender, history of radiotherapy or chemotherapy, *MGMT* promoter methylation, as well as tumor recurrence (**Table 1**). Critically, the expression level of *EVA1C* was significantly and positively correlated with that of vimentin (r = 0.51, *P* = 2.33e-13, **Figure 2A**). These results suggested that high *EVA1C* expression was positively associated with malignant properties of glioma. Furthermore, *EVA1C* was considered as a risk factor for glioma patients as Kaplan-Meier curves showed that the high *EVA1C* expression group presented poorer prognosis than the low expression group (**Figure 2C**).

**Table 1:** The correlation between *EVA1C* expression level and clinicopathological features of patients in the CGGA cohort (n = 182).

Characteristics	*EVA1C* expression		*P* value
	Low expression	High expression	
**Age (years)**			
≥40	53	41	0.075
<40	38	50	
**Sex**			
Male	57	54	0.648
Female	34	37	
**WHO grade**			
WHO II	64	39	<0.0001
WHO III	27	52	
**Histopathology**			
O	41	11	<0.0001
A	27	29	
AO	9	3	
AA	14	48	
**IDH**			
Mutation	87	46	<0.0001
Wildtype	3	45	
**1p/19q**			<0.0001
Codeletion	48	12	
Non-codeletion	42	78	
**MGMT promoter**			
Methylation	52	37	0.066
Unmethylation	34	43	
**Radiotherapy**			
Yes	74	68	0.592
No	15	17	
**Chemotherapy**			
Yes	45	46	0.810
No	39	37	
**Recurrence**			
Yes	15	23	0.145
No	76	68	

O, oligodendroglioma; A, astrocytoma; AO, anaplastic oligodendroglioma; AA, anaplastic astrocytoma.

Next, a larger TCGA cohort of 457 glioma patients was used to validate the above results. Similarly, the analysis results indicated that *EVA1C* expression was correlated with WHO grade, histopathological type, *IDH* mutation status and 1p/19q codeletion (**Supplementary Table 3**). The robust correlation between *EVA1C* and vimentin was also confirmed in the validation cohort (r = 0.657, *P* < 0.0001, **Figure 2B**). Moreover, survival analysis verified the significant association between elevated *EVA1C* expression and poorer prognosis in the TCGA cohort (**Figure 2D**). Additionally, the ROC curves revealed that the AUCs of *EVA1C* for predicting the 1-, 3-, and 5-year survival were 0.810, 0.751, and 0.656, respectively (**Figure 2E**). Finally, we also analyzed the correlation between *EVA1C* expression and the prognosis of patients with other solid tumors, including KIRC, LAML, PAAD and THYM. However, the significant correlation between high *EVA1C* expression and poor prognosis was only observed in patients with LAML (**Supplementary Figure 3**).

### *EVA1C* Was Associated *W*ith Immune-Related Biological Functions

**Figure 3:**
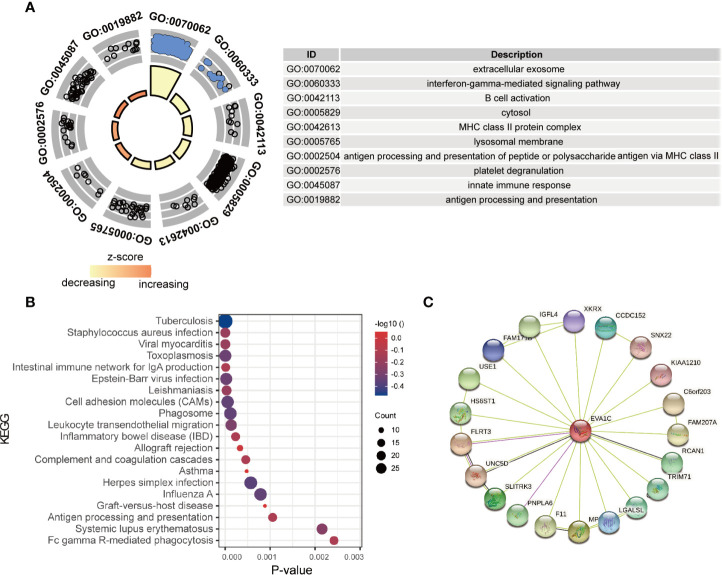
The enrichment analysis of *EVA1C* co-expression genes indicated *EVA1C* was involved in inflammatory and immune biological processes. **(A)** The top 10 GO enrichment terms. **(B)** The top 20 KEGG pathways enriched. **(C)** The protein-protein network was constructed *via the* STRING database.

To identify the potential functions of *EVA1C* in glioma, the top 1000 co-expressed genes of *EVA1C* were extracted and subsequently inputted for enrichment analysis. As shown in **Figure 3A**, genes that co-expressed with *EVA1C* were enriched in several immune-related GO terms, including “innate immune response”, “antigen processing and presentation”, “B cell activation”, and “platelet degranulation”. Meanwhile, the significantly enriched KEGG pathways included “staphylococcus aureus infection”, “viral myocarditis”, “intestinal immune network for IgA production”, “cell adhesion molecules”, “antigen processing and presentation”, and “allograft rejection” (**Figure 3B**). These findings suggested that *EVA1C* might regulate the immune microenvironment through various immune processes such as antigen processing and presentation, complement and coagulation cascades, and intestinal immune network for IgA production. Next, we also used the Coexpedia online website, which based on 384 human GEO datasets and 248 mouse GEO datasets, to analyze the biological functions of *EVA1C* gene. The results also showed that *EVA1C* gene was associated with the activation of NF-KB signaling pathway, macrophage activation involved in immune response, cellular response to interleukin-6 (**Supplementary Figure 4**). Previous study also showed that the activation NF-KB signaling pathway in breast cancer cells could upregulate interleukin-6 expression, and further promote cancer cell metastasis (20).

In addition, the proteins interacting with *EVA1C* (**Figure 3C**) were enriched in terms including ‘complement and coagulation cascades’ and ‘thyroid hormone signaling pathway’, showing the potential role of *EVA1C* in affecting the processes of innate and acquired adaptive immune responses, which might impact tumor initiation and progression.

### Association Between *EVA1C* Expression and Microenvironment of Glioma

**Figure 4:**
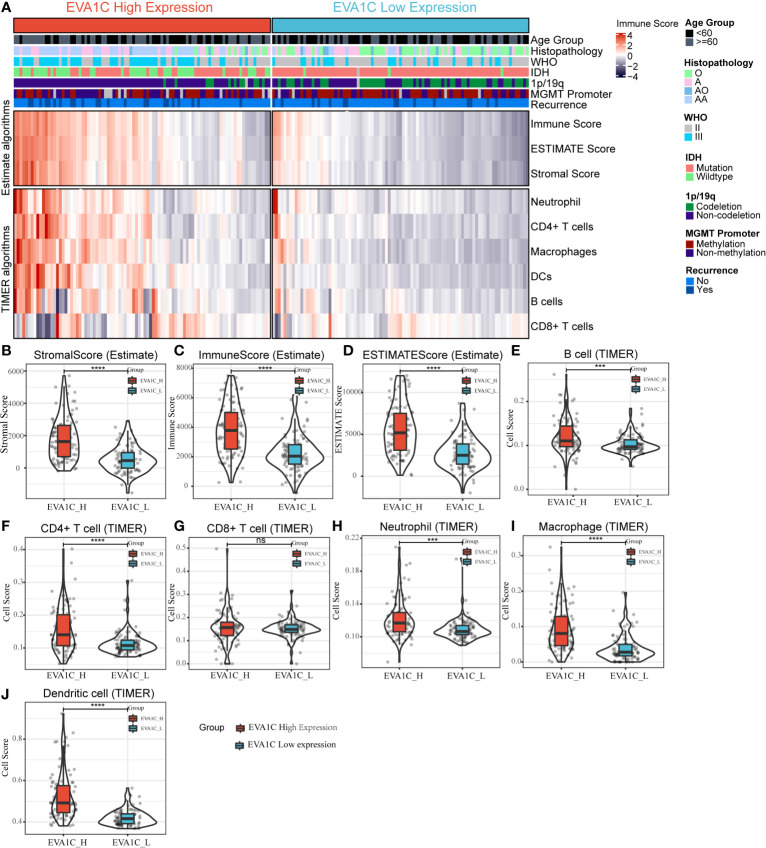
The relationship between *EVA1C* expression and immune infiltration in the CGGA cohort. **(A)** The heatmap represents cell type enrichment score of each immune cell type for the 182 samples. **(B–D)** The comparison of stromal score, immune score, and ESTIMATE score between the high and low *EVA1C* expression groups. **(E–J)** The comparison of the abundance of B cell, CD4+ T cell, CD8+ T cell, neutrophil, macrophage, and dendritic cell between the high and low *EVA1C* expression groups.

**Figure 5:**
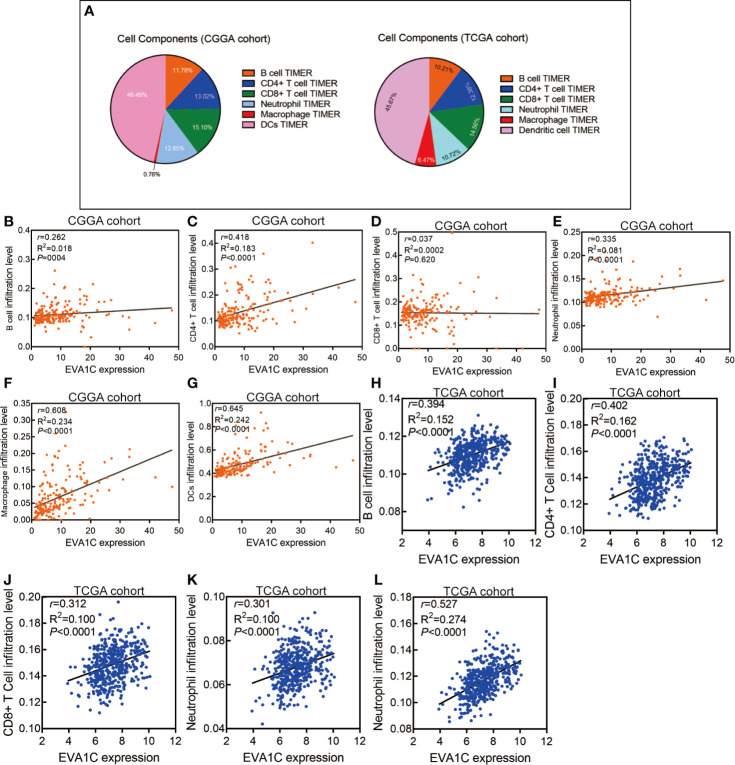
High *EVA1C* expression was positively correlated with immune infiltration levels. **(A)** The heatmap shows the proportions of different immune cell subpopulations in CGGA and TCGA cohorts. The scatter plots show correlations between *EVA1C* expression with the abundance of various immune infiltrates in the CGGA cohort **(B–G)** and the TCGA cohort **(H–L)**.

We used the expression profiler to calculate stromal score, immune score and ESTIMATE score by the ESTIMATE algorithm, and calculate the infiltration abundance of immune cells. The immune landscape illustrated that various immune infiltration levels in the *EVA1C* high expression group were higher than those in the *EVA1C* low expression group (**Figure 4A**). Specifically, the immune score, stromal score, and ESTIMATE score were all significantly higher in the *EVA1C* high expression group (**Figures 4B–D**). Furthermore, we calculated the enrichment scores based on the TIMER algorithm and found that the scores of B cell, CD4+ T cell, neutrophil, macrophage, and DCs (except for CD8+ T cell) in the *EVA1C* high expression group were higher than those in the *EVA1C* low expression group (**Figures 4E–J**). The immune landscape illustrated the proportions of different immune cell subpopulations in CGGA and TCGA cohorts, and the findings were quite consistent (**Figure 5A**). Subsequent scatter plots showed the similar results that *EVA1C* expression had significant correlations with the infiltration levels of B cell (r = 0.262, *P* = 0.004), CD4+ T cell (r = 0.418, *P* < 0.0001), neutrophil (r = 0.335, *P* < 0.0001), macrophage (r = 0.608, *P* < 0.0001), and DCs (r = 0.645, *P* < 0.0001), except for CD8+ T cell (r = 0.037, *P* = 0.620) (**Figures 5B–G**).

**Figure 6:**
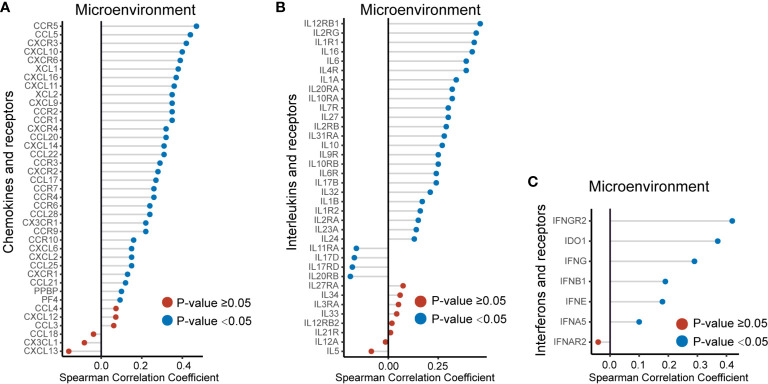
The *EVA1C* expression was correlated with the expression of cytokines, including chemokines **(A)**, interleukins **(B)** and interferons **(C)**, and their receptors in the microenvironment of WHO grade II/III glioma.

We also used the TCGA validation cohort to verify the above positive correlations. Similar results were obtained that elevated *EVA1C* expression was associated with higher abundance of various immune infiltrates (**Supplementary Figure 5**). Likewise, significant correlations were observed between *EVA1C* expression and infiltration levels of B cell (r = 0.394, *P* < 0.0001), CD4+ T cell (r = 0.402, *P* < 0.0001), CD8+ T cell (r = 0.312, *P* < 0.0001), neutrophil (r = 0.301, *P* < 0.0001), and macrophage (r = 0.527, *P* < 0.0001) in the TCGA validation cohort (**Figures 5H–L**). These findings strongly indicated the important role *EVA1C* played in immune infiltrating processes in the context of WHO grade II/III glioma. Finally, the correlation between *EVA1C* expression and the mRNA levels of chemokines, interleukins, interferons and other important cytokines and their receptors in the microenvironment of WHO grade II/III glioma by GEPIA database (**Figure 6**). These results suggested that, in the microenvironment of glioma with high *EVA1C* expression, there were not only a variety of immune cells, but also high expression of many chemokines including *CCR5, CCL5, CXCL10*, and *CXCL9*, which have been shown to attract DCs, T cell.

### Correlation Analysis Between *EVA1C* Expression and Immune Marker Sets

**Figure 7:**
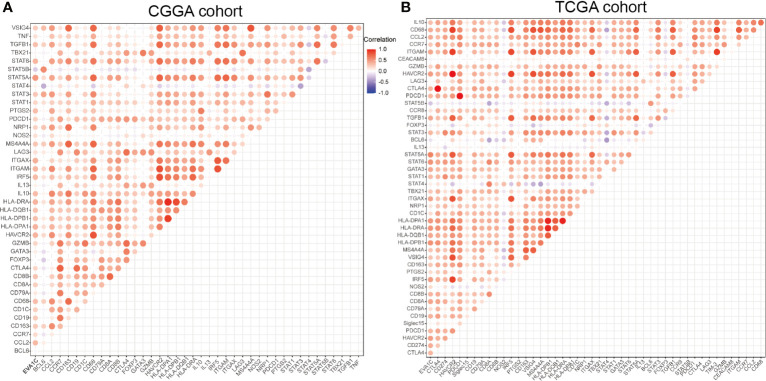
Correlation between *EVA1C* and marker gene sets of immune cells in the CGGA cohort **(A)** and the TCGA cohort **(B)**.

Given the correlation between *EVA1C* expression and immune infiltration levels, we further analyzed the relationship between *EVA1C* expression and the marker genes of essential immune cells in glioma. **Figure 7A** shows the correlations between *EVA1C* expression and various immune markers in the CGGA cohort. Interestingly, *EVA1C* expression was associated with gene markers (21) of B cell, CD8+ T cell, M2 macrophage, DCs, Th2 cell, exhausted T cell, and neutrophil in WHO II/III glioma (**Supplementary Table 4**). These results suggested that *EVA1C* might play a specific role in regulating macrophage polarization in WHO II/III glioma. In addition, *EVA1C* expression was related to the markers of tumor associated macrophage (TAM), such as CCL2, CD68 and IL10. These findings further revealed a robust interaction between *EVA1C* and TAM infiltration. Furthermore, a significant relationship was detected between *EVA1C* expression and DCs markers (*HLA-DPB1, HLA-DRA, HLA-DPA1, CD1C* and *ITGAX*). In addition, significant correlations were found between *EVA1C* and TGFβ (TGFB1, marker of Treg cell), as well as TIM-3 (HAVCR2, T cell exhaustion) (**Supplementary Table 4**). These results were further confirmed in the TCGA validation cohort (**Figure 7B**), suggesting that *EVA1C* participated in immune escape within the tumor microenvironment of WHO II/III glioma.

### The Performance of *EVA1C* in Predicting a High Immune Score in Glioma

**Figure 8:**
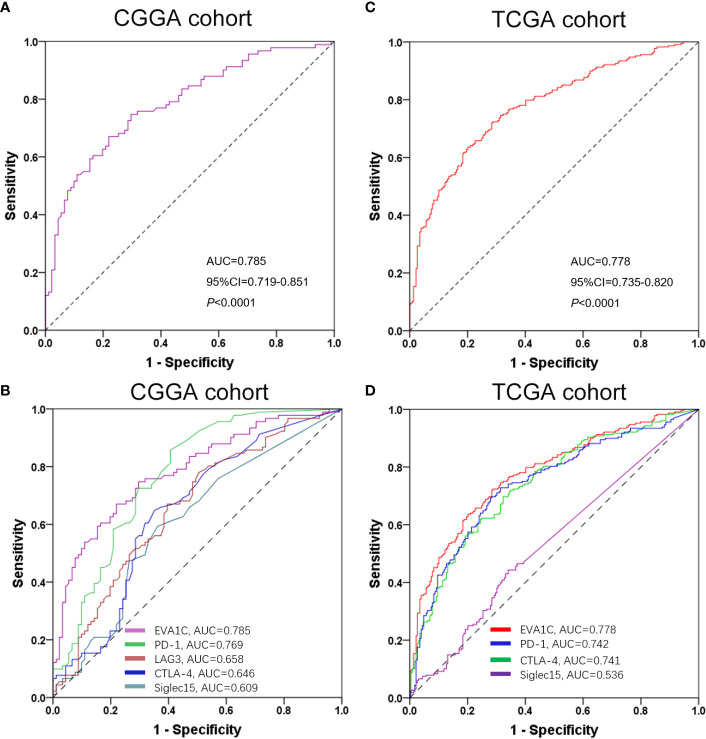
ROC curves of *EVA1C* gene for predicting high immune infiltration showed excellent power. ROC curves of *EVA1C* and other indicators in predicting high immune infiltration levels in the CGGA cohort **(A, B)** and the TCGA cohort **(C, D)**.

To determine whether *EVA1C* could be considered as a potential biomarker to discriminate the immune infiltration levels in glioma, we applied the ROC curve to evaluate the ability of *EVA1C* in predicting a high immune score for glioma. As shown by **Figure 8A**, the sensitivity and specificity of *EVA1C* in predicting a high immune score in the CGGA cohort were 74.7% and 70.3%, respectively. The area under the curve (AUC) was 0.785. Additionally, we also compared the predictive performance between *EVA1C* and other commonly utilized indicators including PD-1, LAG3, CTLA-4 and Siglec15. In terms of AUC, *EVA1C* demonstrated the highest predictive performance in predicting a high immune score within glioma (**Figure 8B**). The consistent results were obtained in the TCGA cohort (**Figures 8C, D** **)**.

## Discussion

Nowadays, immune-oncological microenvironment has become the focus of cancer researches (22). Immunosuppressant began to be gradually applied to clinical patients. Although PD-1 and CTLA-4 antibodies have achieved a sustained response in some patients (23), most patients with glioma demonstrated poor responses to them. Such phenomena could be attributed to the essential and complicated immune escape processes in tumor microenvironment (24). There are multiple macrophages infiltrating in the glioma TME, which could prevent the immune system from eliminating malignant cells effectively (25). Thus, a deepening understanding of the interplay between TME and immunotherapy not only helps to explore the mechanism of immune escape, but also provides new approaches to improve the immunotherapeutic efficacy.

In this study, we observed for the first time that *EVA1C* was significantly overexpressed in glioma, and significantly correlated with malignant clinicopathological features. In this study, *EVA1C* high expression might be a potential poor prognostic factor. KEGG and GO enrichment analyses showed that *EVA1C* expression correlated with immune and inflammation related biological processes.

In mammals, the EVA1 family mainly includes three members: *EVA1A, EVA1B* and *EVA1C*. Previous studies showed that *EVA1A* (*TMEM166*) protein which was located in cell membrane could induce cell autophagy and apoptosis (26, 27). Ming Tao et al. found that, compared with normal pancreatic acinar cells, the *EVA1C* expression was remarkably higher in pancreatic acinar carcinoma, and it was mainly located in cell membrane and cytoplasm (28). Interestingly, Ziyi Wang et al. reported that *EVA1A* could inhibit *NLRP3* activation to reduce liver hypoxia-reperfusion damage *via* inducing autophagy in Kupffer cells (29). On the other hand, Bang-Yi Lin et al. found that *EVA1A* promoted papillary thyroid cancer progression and EMT by the Hippo signaling pathway (30). As a core kinase of the Hippo signaling pathway in cancers, *MST1/2* participates in the development and function of Treg and Th17 cells (31). The imbalance of these two cell types is a leading cause of multiple inflammatory and autoimmune diseases (32). The *EVA1C* is mainly mapped to critical region chromosome 21 (21q21-21q22.3) associated with Down syndrome. Intriguingly, Gregory James et al. found that the expression pattern of *EVA1C* was consistent with an axon guidance role in the mouse nervous system (33). However, what is the role of EVA1C plays in glioma is not reported. This is a subject that needs to be further explored.

A key finding in our study is that high *EVA1C* expression correlates with the high abundance of immune infiltrates including B cells, CD4+ T cells, neutrophils, macrophages and DCs in WHO grade II/III glioma. There is no statistical correlation between EVA1C and CD8+ T cell infiltration level in CGGA cohort. The possible reason is low statistical power due to small sample size, which need to further explore. Significant correlations between the *EVA1C* and the markers of TAMs as well as M2 macrophages suggest that *EVA1C* possesses the potential in regulating the polarization of TAMs, which is crucial for cancer development and metastasis (34). Adam Wu et al. found that cancer stem cells promote immunosuppression by M2 macrophages secreting many kinds of cytokines, such as TGF-β1 and IL-10 (35). IL-10 and TGF- β 1 has been shown to promote tumor cell immune escape by inhibiting T cell proliferation (36). Another intriguing finding in our study is the relationship between *EVA1C* and immune markers of DCs, Treg cells, and exhausted T cells. DCs can induce tumor cell metastasis by favoring Treg cells and reducing CD8+ T cell cytotoxicity (37). Additionally, TGF-β secreted by M2 macrophages could induce the shift from immature CD4+ T cells to Treg cells, and promote the proliferation of them (38). These outcomes reveal that *EVA1C* protein may increase the recruitment of immune cells in WHO grade II/III glioma. Notably, the predictive performance of *EVA1C* in predicting high immune infiltration levels was excellent, demonstrating its potential in predicting immune profiles in WHO II/III glioma. However, it is still unclear whether the expression of *EVA1C* is related to the efficacy of immunotherapy and chemotherapy for glioma, and there is no data in this regard at present.

Although we revealed an immune-related biomarker and target for the first time in the patients with glioma, this study has some limitations. Firstly, the aim of this study was to elaborate the findings from the perspective of genomics, and the analysis of gene transcription levels could only reflect some aspects of immune status, but not the overall changes. In this study, Estimate and Timer algorithms were adopted, and conventional statistical methods were used for analysis. Secondly, although the above results could be validated in TCGA cohort with 457 patients, they need to be verified by another retrospective single-center cohort. Thirdly, the functions and in-depth mechanisms of *EVA1C* were explored *in vitro*. This study is only an exploratory discovery, and lays a foundation for our next functional mechanism experiments.

In summary, we found that *EVA1C* expression was upregulated in glioma compared with normal brain tissues, and the elevated expression level was significantly associated with malignant features and poor prognosis of glioma patients. Importantly, high *EVA1C* expression correlated with high immune infiltration levels and chemokines, interleukins, interferons and their receptors in WHO grade II/III glioma. In addition, *EVA1C* expression was significantly correlated with gene expression of M2 macrophages, TAMs, DCs, exhausted T cells and Treg cells markers. These findings suggest that *EVA1C* may be not only a potential immune-related biomarker, but also a key modulator in governing tumor microenvironment.

## Data Availability Statement

Publicly available datasets were analyzed in this study. This data can be found here: Chinese Glioma Genome Atlas (CGGA, http://www.cgga.org.cn/) and The Cancer Genome Atlas (TCGA, https://tcga-data.nci.nih.gov/tcga/) databases.

## Ethics Statement

The studies involving human participants were reviewed and approved by the Ethics Committee of Nanfang Hospital. The ethics committee waived the requirement of written informed consent for participation.

## Author Contributions

Conception and design: SQ and ZH. Data analysis: SQ. Writing and revising: ZH. All authors contributed to the article and approved the submitted version.

## Conflict of Interest

The authors declare that the research was conducted in the absence of any commercial or financial relationships that could be construed as a potential conflict of interest.
