# Supplementary material for: EVA1C Is a Potential Prognostic Biomarker and Correlated With Immune Infiltration Levels in WHO Grade II/III Glioma
Source: Front Immunol. 2021 Jun 29;12:683572. doi: 10.3389/fimmu.2021.683572 (PMC8277382; doi:10.3389/fimmu.2021.683572)
Supplement: Supplementary file 1 [file DataSheet_1.docx]

**
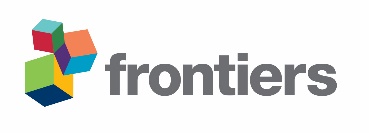
**

**Supplementary Material**

1. Supplementary Figures and Tables

1.1 Supplementary Figures


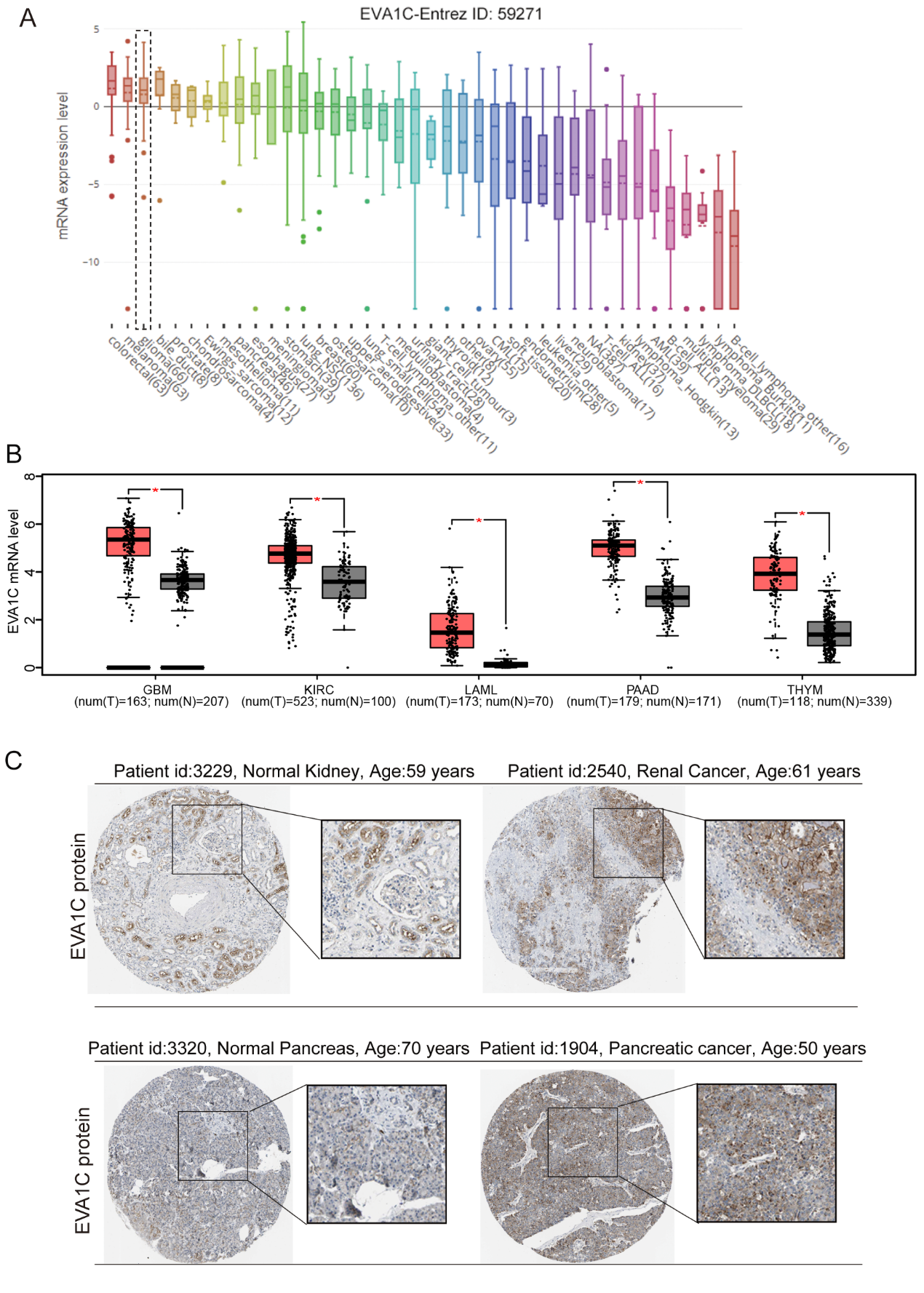


**Supplementary FIGURE 1 ⎜** The *EVA1C* mRNA and protein levels in other kinds of cancers. (**A**) The mRNA expression levels of *EVA1C* in multiple cancer cell lines according to the CCLE database. (**B**) *EVA1C* mRNA levels were upregulated in KIRC, LAML, PAAD and THYM (GEPIA website). (**c**) Compared with those in the normal tissues, the *EVA1C* protein levels in renal cancer and pancreatic cancer were upregulated. T: Tumor tissues; N: Normal tissues.


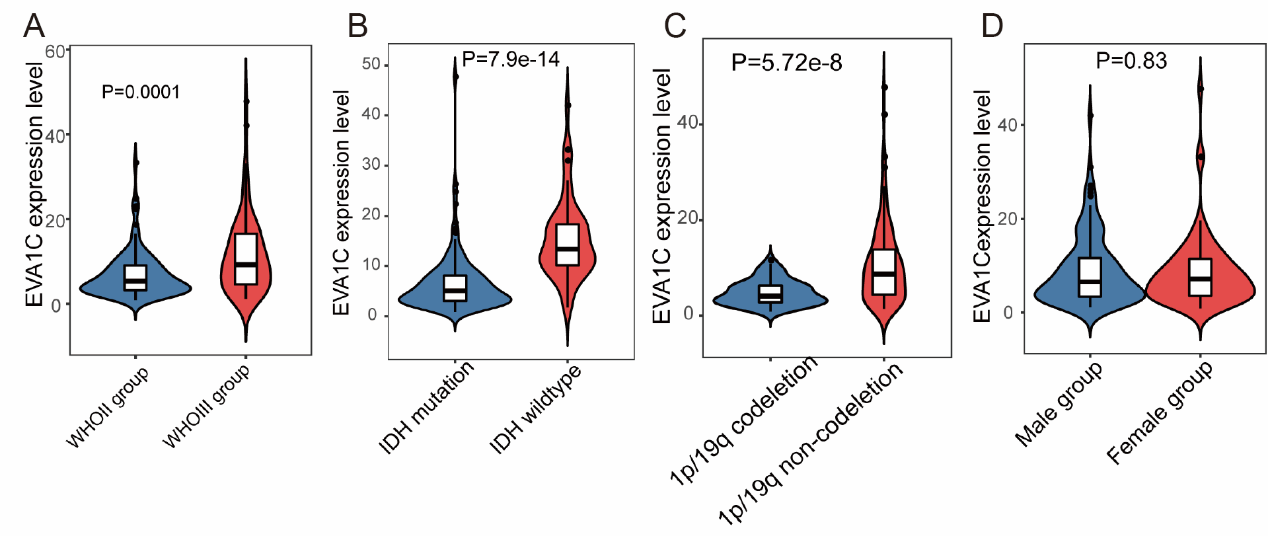


**Supplementary FIGURE 2 ⎜** *EVA1C* expression was correlated with clinicopathological factors, including WHO grade (**A**), IDH status (**B**), 1p/19q status (**C**) and gender (**D**).


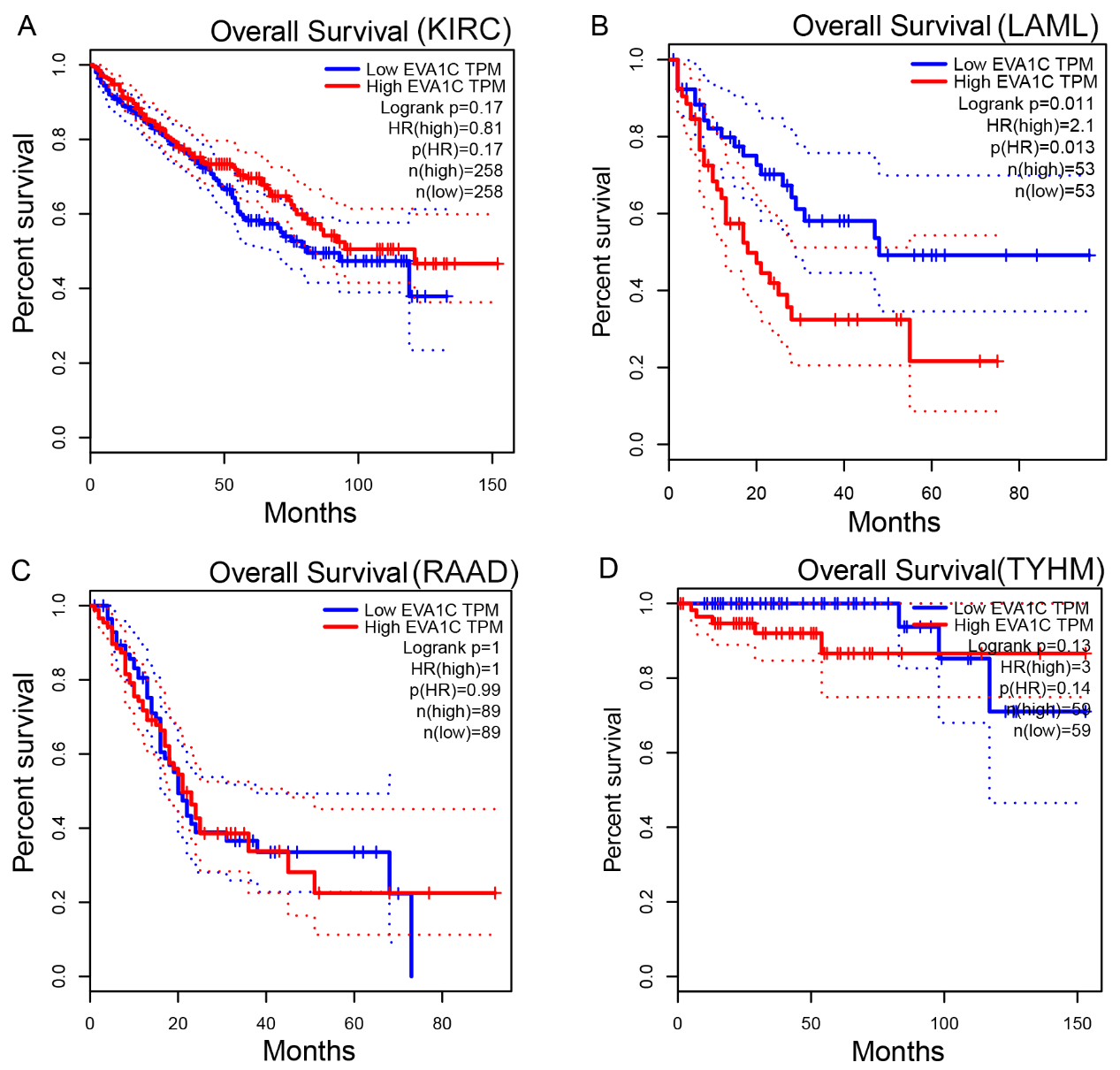


**Supplementary FIGURE 3 ⎜** Kaplan-Meier survival curves of patients with low and high expression of *EVA1C* in other four kinds of tumors including KIRC, LAML, RAAD and TYHM. (**A-D**) The Kaplan-Meier curve shows that higher *EVA1C* expression is not associated with poor prognosis, except for LAML.


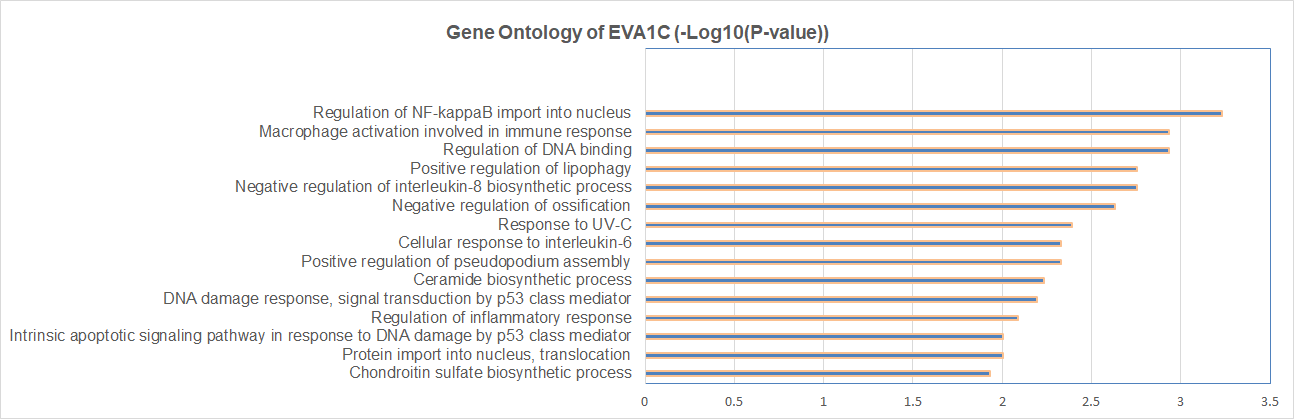


**Supplementary FIGURE 4 ⎜** Gene Ontology Enrichment analysis (biological process) of *EVA1C* using Coexpedia internet tool that was based on public GEO datasets.


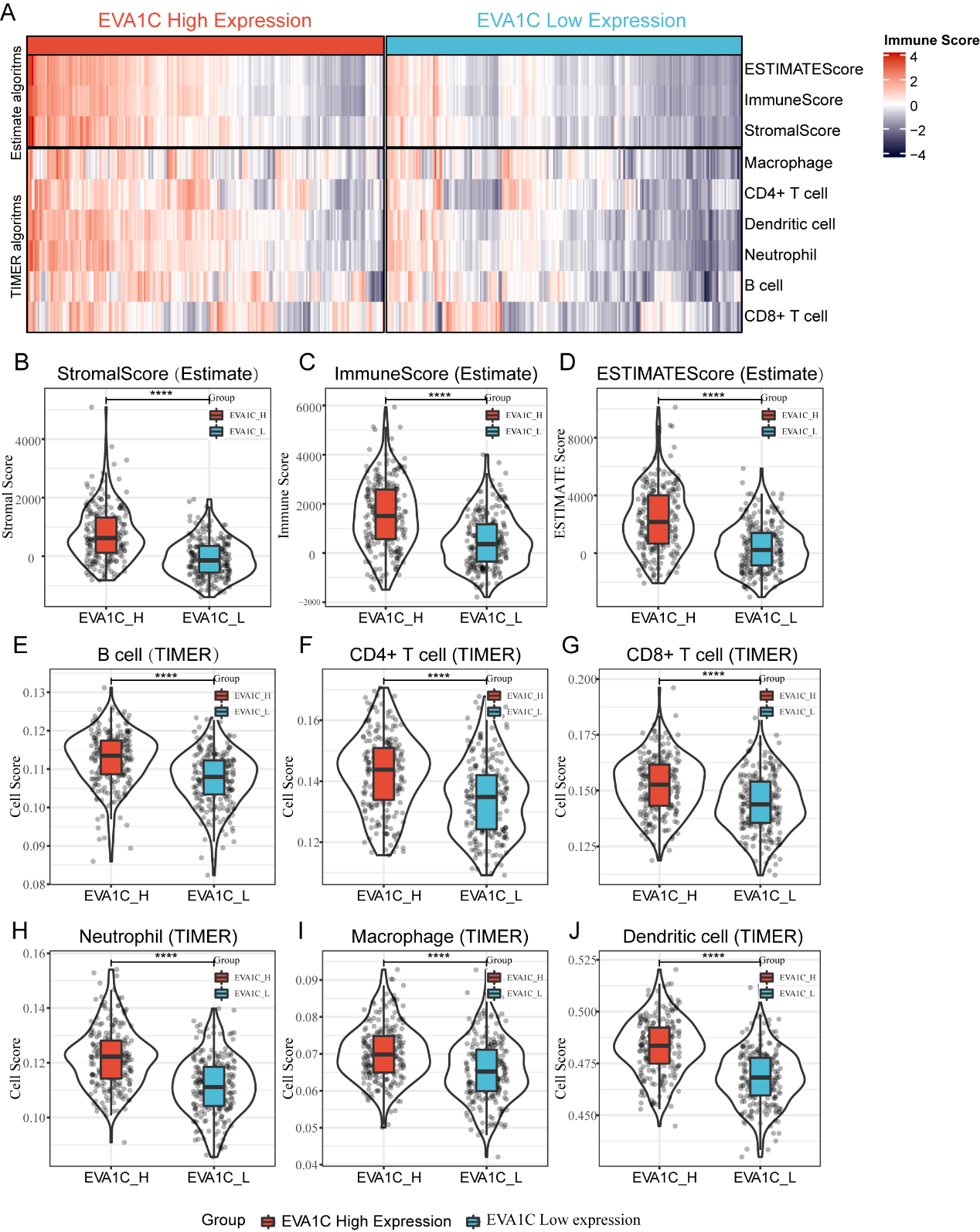


**Supplementary FIGURE 5 ⎜** The validation of correlation between *EVA1C* expression and immune cell populations in the TCGA cohort. (**A**) The heatmap represents cell type enrichment score of each immune cell type for the 457 samples. (**B-J**) The comparison of stromal score, immune score, ESTIMATE score, B cell, CD4+ T cell, CD8+ T cell, neutrophil, macrophage, and dendritic cell between high and low *EVA1C* expression groups.

- 1. Supplementary Tables

Supplementary Table 1. Clinicopathological characteristics of patients (CGGA cohort).

| **Characteristics** | **Category** | **Number of cases** | **(%)** |
| --- | --- | --- | --- |
| **Age(years)** | <40 | 94 | 51.6 |
|  | ≥40 | 88 | 48.4 |
| **Sex** | Male | 111 | 61.0 |
|  | Female | 71 | 39.0 |
| **WHO grade** | WHOII | 103 | 56.6 |
|  | WHOIII | 79 | 43.4 |
| **Histopathology** | O | 52 | 28.6 |
|  | A | 56 | 30.8 |
|  | AO | 12 | 6.6 |
|  | AA | 62 | 34.1 |
| **IDH** | Mutation | 133 | 73.1 |
|  | Wildtype | 48 | 26.4 |
|  | NA | 1 | 0.5 |
| **1p/19q** | Codel | 60 | 33.0 |
|  | Non-codel | 120 | 65.9 |
|  | NA | 2 | 1.1 |
| **MGMT promoter methylation** | Yes | 89 | 48.9 |
|  | No | 77 | 42.3 |
|  | NA | 16 | 8.8 |
| **Radiotherapy** | Yes | 142 | 78.0 |
|  | No | 32 | 17.6 |
|  | NA | 8 | 4.4 |
| **Chemotherapy** | Yes | 91 | 50.0 |
|  | No | 76 | 41.8 |
|  | NA | 15 | 8.2 |
| **Recurrence** | Yes | 38 | 20.9 |
|  | No | 144 | 79.1 |

O: oligodendroglioma; A: astrocytoma; AO: anaplastic oligodendroglioma; AA: anaplastic astrocytoma.

Supplementary Table 2. Clinicopathological characteristics of patients (TCGA cohort).

| **Characteristics** | **Category** | **Number of cases** | **(%)** |
| --- | --- | --- | --- |
| **Age(years)** | <40 | 212 | 46.4 |
|  | ≥40 | 245 | 53.6 |
| **Sex** | Male | 256 | 56.0 |
|  | Female | 201 | 44.0 |
| **WHO grade** | WHOII | 216 | 47.3 |
|  | WHOIII | 241 | 52.7 |
| **Histopathology** | O | 117 | 25.6 |
|  | OA | 44 | 9.6 |
|  | A | 55 | 12.0 |
|  | AO | 87 | 19.0 |
|  | AOA | 40 | 8.8 |
|  | AA | 114 | 24.9 |
| **IDH** | Mutation | 369 | 18.8 |
|  | Wildtype | 86 | 80.7 |
|  | NA | 2 | 0.4 |
| **1p/19q** | Codel | 151 | 33.0 |
|  | Non-codel | 306 | 67.0 |

O: oligodendroglioma; OA: oligoastrocytoma; A: astrocytoma; AO: anaplastic oligodendroglioma; AOA: anaplastic oligoastrocytoma; AA: anaplastic astrocytoma.

Supplementary Table 3. The correlation between *EVA1C* expression and clinicopathological features in TCGA cohort (n=457).

| **Characteristics** | **EVA1C expression** | |  |
| --- | --- | --- | --- |
|  | **Low expression** | **High expression** | ***P*-value** |
| **Age** |  |  |  |
| ≥40 | 116 | 96 | 0.055 |
| ＜40 | 112 | 133 |  |
| **Sex** |  |  |  |
| Male | 127 | 129 | 0.892 |
| Female | 101 | 100 |  |
| **WHO grade** |  |  |  |
| WHO II | 120 | 96 | 0.022 |
| WHO III | 108 | 133 |  |
| **Histopathology** |  |  |  |
| O | 81 | 36 | <0.0001 |
| OA | 21 | 23 |  |
| A | 18 | 37 |  |
| AO | 59 | 28 |  |
| AOA | 11 | 29 |  |
| AA | 38 | 76 |  |
| **IDH** |  |  |  |
| Mutation | 224 | 145 | <0.0001 |
| Wildtype | 4 | 82 |  |
| **1p/19q** |  |  |  |
| Codel | 119 | 32 | <0.0001 |
| Non-codel | 109 | 197 |  |

O: oligodendroglioma; OA: oligoastrocytoma; A: astrocytoma; AO: anaplastic oligodendroglioma; AOA: anaplastic oligoastrocytoma; AA: anaplastic astrocytoma

Supplementary Table 4. Correlation between *EVA1C* expression and marker genes of different immune cells in CGGA cohort (n=182).

| Description | Marker Genes | Correlation Coefficient | P-value |
| --- | --- | --- | --- |
| B cell | CD19 | 0.32 | *** |
|  | CD79A | 0.27 | *** |
| CD8+ | CD8A | 0.4 | *** |
|  | CD8B | 0.29 | *** |
| M1 | NOS2 | 0.28 | *** |
|  | IRF5 | 0.44 | *** |
|  | PTGS2 | 0.3 | *** |
| M2 | CD163 | 0.56 | *** |
|  | VSIG4 | 0.48 | *** |
|  | MS4A4A | 0.54 | *** |
| DCs | HLA-DPB1 | 0.61 | *** |
|  | HLA-DQB1 | 0.52 | *** |
|  | HLA-DRA | 0.62 | *** |
|  | HLA-DPA1 | 0.57 | *** |
|  | CD1C | 0.3 | *** |
|  | NRP1 | 0.57 | *** |
|  | ITGAX | 0.33 | *** |
| Th1 | TBX21 | 0.36 | *** |
|  | STAT4 | -0.089 | 0.029 |
|  | STAT1 | 0.36 | *** |
|  | TNF | -0.04 | 0.34 |
| Th2 | GATA3 | 0.49 | *** |
|  | STAT6 | 0.42 | *** |
|  | STAT5A | 0.51 | *** |
|  | IL13 | 0.038 | 0.352 |
| Tfh | BCL6 | -0.26 | *** |
|  | IL21 | 0.18 | **** |
| Th17 | STAT3 | 0.39 | *** |
|  | IL17A | -0.01 | 0.85 |
| Treg | FOXP3 | -0.04 | 0.26 |
|  | TGFB1 | 0.67 | *** |
|  | CCR8 | 0.29 | *** |
|  | STAT5B | -0.55 | *** |
| T cell exhaustion | PDCD1 | 0.5 | *** |
|  | CTLA4 | 0.37 | *** |
|  | LAG3 | 0.33 | *** |
|  | HAVCR2 | 0.52 | *** |
|  | GZMB | 0.47 | *** |
| Neutrophils | CEACAM8 | 0.12 | *** |
|  | ITGAM | 0.35 | *** |
|  | CCR7 | 0.43 | *** |
| TAMs | CCL2 | 0.53 | *** |
|  | CD68 | 0.55 | *** |
|  | IL10 | 0.55 | *** |
